# Role of crotoxin in coagulation: novel insights into anticoagulant mechanisms and impairment of inflammation-induced coagulation

**DOI:** 10.1590/1678-9199-JVATITD-2020-0076

**Published:** 2020-11-27

**Authors:** Bruna Terada Gimenez, Gabriel Neves Cezarette, Aline de Sousa Bomfim, Wuelton Marcelo Monteiro, Elisa Maria de Sousa Russo, Fabiani Gai Frantz, Suely Vilela Sampaio, Marco Aurelio Sartim

**Affiliations:** 1 Department of Clinical Analysis, Toxicology and Food Science, School of Pharmaceutical Sciences of Ribeirão Preto, University of São Paulo (USP), Ribeirão Preto, SP, Brazil.; 2 Center for Cell-Based Therapy and Regional Blood Center of Ribeirão Preto, University of São Paulo (USP), Ribeirão Preto, SP, Brazil.; 3 Tropical Medicine Graduate Program, Amazonas State University, Manaus, AM, Brazil.; 4 Carlos Borborema Clinical Research Institute, Doutor Heitor Vieira Dourado Tropical Medicine Foundation, Manaus, AM, Brazil.; 5 Basic and Applied Immunology Graduate Program, Institute of Biological Sciences, Federal University of Amazonas, Manaus, AM, Brazil.

**Keywords:** Crotoxin, Anticoagulant mechanism, Phospholipase A_2_, Anti-inflammatory activity, Snake venom, Tissue factor, Coagulation factors, Coagulation complex, Coagulation phospholipids, Cytokines

## Abstract

**Background:**

Snake venom phospholipases A_2_ (svPLA_2_) are biologically active toxins, capable of triggering and modulating a wide range of biological functions. Among the svPLA_2_s, crotoxin (CTX) has been in the spotlight of bioprospecting research due to its role in modulating immune response and hemostasis. In the present study, novel anticoagulant mechanisms of CTX, and the modulation of inflammation-induced coagulation were investigated.

**Methods:**

CTX anticoagulant activity was evaluated using platelet poor plasma (PPP) and whole blood (WB), and also using isolated coagulation factors and complexes. The toxin modulation of procoagulant and pro-inflammatory effects was evaluated using the expression of tissue factor (TF) and cytokines in lipopolysaccharide (LPS)-treated peripheral blood mononuclear cells (PBMC) and in WB.

**Results:**

The results showed that CTX impaired clot formation in both PPP and WB, and was responsible for the inhibition of both intrinsic (TF/factor VIIa) and extrinsic (factor IXa/factor VIIIa) tenase complexes, but not for factor Xa and thrombin alone. In addition, the PLA_2_ mitigated the prothrombinase complex by modulating the coagulation phospholipid role in the complex. In regards to the inflammation-coagulation cross talk, the toxin was capable of reducing the production of the pro-inflammatory cytokines IL-1β, IL-6 and TNF-α, and was followed by decreased levels of TF and procoagulant activity from LPS-treated PBMC either isolated or in WB.

**Conclusion:**

The results obtained in the present study recognize the toxin as a novel medicinal candidate to be applied in inflammatory diseases with coagulation disorders.

## Background

Snake venom phospholipases A_2_ (svPLA_2_) are enzymes that when secreted catalyze the hydrolysis of phospholipids. These enzymes are responsible for local and systemic effects such as myotoxicity, neuromuscular blockade, inflammation, and hemostasis alterations [1-4]. Aside from the toxicological behavior presented by this group of toxins, their wide range of pharmacological properties have brought novel perspectives for svPLA_2_s as antitumoral, analgesic, bactericidal, immunosuppressive and anticoagulant agents [4-8]. 

Several svPLA_2_ have been described to modulate hemostasis events, upon which anticoagulant behavior has been widely reported. The inhibition of the blood coagulation cascade involves the impairment of the formation of coagulation complexes, which are composed of clotting factors, cofactors, ions and phospholipids. The mechanisms postulated for svPLA_2_ comprise i) the hydrolysis of procoagulant phospholipids; ii) the competition with coagulation factor for phospholipid binding, and/or iii) directly binding to coagulation factors thus preventing complex formation. Whether it is one or all of these, the overall response is the prevention of fibrin formation [9,10]. 

Crotoxin (CTX) is a protein complex from the venom of the South American rattlesnake *Crotalus durissus terrificus* (Cdt), and is composed of a basic enzymatically active asp49 PLA_2_ (CB) and an acidic non-enzymatic domain (CA). The toxin has been extensively investigated due to its major role as the main toxic component of Cdt venom [11]. However, several pharmacological properties have been reveled over the past 30 years, such as anticoagulant activity. As in many svPLA_2_s, both CB and Crotoxin complex (CB/CA) are capable of inhibiting prothrombinase complex formation through direct interaction with factor Xa (FXa) [12]. Another interesting pharmacological aspect of CTX, that differentiates it from other svPLA_2_ anticoagulants, concerns its immune modulation properties. The toxin is capable of modulating cellular events of both innate and humoral immunity, resulting in a immunosuppressive and anti-inflammatory response that involves the production of pro-resolving lipid mediators, such as lipoxin A_4_ [6]. 

Coagulopathy is a condition that is either inherited, congenital or acquired, and is characterized by the imbalance of hemostatic events that results in thrombotic and/or bleeding disorders [13]. The disseminated intravascular coagulation (DIC) is a common complication in sepsis, and represents a relevant case of inflammation-induced coagulopathy. The major pathophysiological mechanism associated with the inflammation/coagulation crosstalk in DIC involves the intravascular expression of tissue factor (TF - coagulation factor III) by endothelial cells and monocytes elicited to the inflammatory site, which triggers the coagulation cascade by forming the extrinsic tenase complex (TF/factor VII) [14,15]. 

The therapeutic approach for treatment of coagulopathies with an inflammatory background relies not only on agents with anticoagulant properties, but also with anti-inflammatory effects [16,17]. Considering CTX’s capacity to modulate inflammation and coagulation, in the present study novel aspects on the anticoagulant mechanism of the toxin were investigated. Moreover, we evaluated CTX’s anti-inflammatory property regarding cultured peripheral blood mononuclear cells (PBMC) and whole blood that may impair TF-mediated procoagulant activity.

## Methods

### Crotoxin

Crotoxin (CTX) was isolated from *Crotalus durissus terrificus* venom, obtained from male and female adult specimens from the serpentarium of the Central Animal Facility of University of São Paulo, Ribeirão Preto. The purification was performed according to Muller [18]. Purified CTX was submitted to Affi-Prep Polymyxin Resin (Bio-Rad, Hercules, USA), according to the manufacturer’s instructions, in order to remove endotoxin contaminants, whose levels were lower than 0.01 EU/µg of CTX (1 EU = 0.1 ng of endotoxin), and which was determined using the limulus amoebocyte lysate kit (Lonza Biosciences, Walkersville, USA). Protein quantification was performed using a BCA kit (Thermo Scientific, Rockford, USA), according to the manufacturer’s instructions.

### Whole blood, platelet poor plasma and PBMC

Anticoagulated blood was obtained from six healthy donors (male and female with ages ranging from 20 to 40 years), who had not received immunological or hemostatic therapy during the last one month, in heparin (143 U/10mL of blood) and sodium citrate (3.2%) tubes.

Platelet poor plasma (PPP) was obtained from sodium citrated blood centrifuged at 1125 *g* for 20 min at room temperature. A pool with the six PPPs was prepared and stored at -80°C for coagulation assays. The pool was assessed for prothrombin time (PT) and partial activated thromboplastin time (aPTT) in an independent routine clinical laboratory, using the reagents Coagulação TP and Coagulação TTPa (WAMA diagnostic, São Carlos, Brazil) in a Coagmaster 4.0 coagulometer (WAMA diagnostic, São Carlos, Brazil). The pool presented normal values (26.4 seconds for aPTT - reference 24 to 39 seconds/12.9 seconds for TP - reference 12 to 15 seconds/INR = 1.03 - reference ~1.0).

Peripheral blood mononuclear cells (PBMC) were obtained by density gradient centrifugation from heparinized blood using Histopaque 1077 (Sigma-Aldrich, St. Louis, USA), according to the manufacturer’s instructions. 

The study protocol was approved by the Research Ethics Committee of School of Pharmaceutical Sciences of Ribeirão Preto, USP (CEP-FCFRP) (CAAE: 90173018.0.0000.5403).

### Plasma coagulation assays: prothrombin time and partial activated thromboplastin time

 PT and aPTT assays were performed using 96-well plates, and the method was adapted from the manufacturer’s instructions. Considering the time between placing the reagents and reading in this technique, both reagents from PT and aPTT containing the agonists (thromboplastin and cephalin+ellagic acid, respectively) were diluted 1:20 (v/v) in saline (0.9% NaCl) in order to purposefully reduce the coagulation velocity and increase clotting time. For PT, 25 µL of CTX (0.1-12.5 µg/mL final reaction concentration) or 25 µL of PBS (control) was incubated with 150 µL of PPP (sodium citrate) for 10 minutes. After the incubation, 25 µL of regent containing thromboplastin (diluted 1:20 v/v; Wiener Lab, Rosário, Argentina) and 25 µL CaCl_2_ (250 mM) were added. For the aPTT, 25 µL of CTX (0.1-12.5 µg/mL final reaction concentration) or 25 µL of PBS (control) was incubated with 150 µL of PPP for 10 minutes, following addition of 25 µL of regent containing cephalin and ellagic acid (diluted 1:20; Wiener Lab, Rosário, Argentina). After 3 minutes, 25 µL CaCl_2_ (250mM) was also added. After the addition of calcium, the clotting time was recorded using a Spectramax 190 microplate reader (Molecular Devices, San Jose, USA). The clotting time was calculated as ½ of Vmax from the clotting curve from sequential readings at 405 nm using SoftMax Pro 6.2 software (Molecular Devices, San Jose, USA). The entire procedure was performed at 37°C.

CTX’s anticoagulant activity in whole blood was also evaluated, using fresh blood collected in sodium citrate. CTX (0.5-12.5 µg/mL) was incubated with whole blood (500 µL) for 10 minutes at 37°C. Then, the material was centrifuged at 1125 *g* for 20 minutes and PPP was thus obtained. Plasma coagulation using both PT and aPTT was performed as described above.

### Staclot^®^ DRVV assay

In the present study we evaluated the toxin’s capacity to modulate the role of coagulation phospholipids (PL) in the prothrombinase complex activity, using the Staclot^®^ DRVV kit (Diagnostica Stago, Asnières-sur-Seine, France). The kit is applied in the diagnosis of lupus anticoagulants, which are heterogenous autoantibodies that target the epitopes of the prothrombinase complex and mitigate its activity by impairing PL binding to FXa. The kit is composed of two reagents (STA-Staclot DRVV Screen and STA Staclot DRVV Confirm), both containing diluted Russel’s viper venom that activates coagulation factor X (FX) into FX activated (FXa) in order to induce plasma clot. The difference between the two reagents is the concentration of the PL: the STA-Staclot DRVV Screen presents low PL content, while the STA Staclot DRVV Confirm utilizes high levels of PL.

 PPP (150 µL) was incubated with 25 µL of CTX (1.5-13.5 µg/mL final reaction concentration) or PBS (control) for 10 min at 37°C in 96-well plate. After, 100 µL of Screen or Confirm reagents (diluted 1:3 v/v in saline) were added and coagulation time recorded as described before.

### Factor Xa and thrombin activity

The modulation of FXa and thrombin activity was evaluated by colorimetric assay. Briefly, CTX (0.5-13.5 µg/mL) or PBS (control) was incubated with 43 mU/mL of human FXa or thrombin (Sigma-Aldrich, St. Louis, USA) for 10 minutes. Afterwards, specific chromogenic substrate S-2222 or S-2238 (400 µM, Chromogenix, Milan, Italy), were added and the hydrolysis of chromogenic substrate by FXa or thrombin was measured at 405 nm using a Spectramax 190 microplate reader (Molecular Devices, San Jose, USA). The reaction was carried out in a 96-well plate at 25ºC in PBS, pH 7.4. The enzymatic activity was calculated based on the slope of the activity curve, obtained from sequential readings at 405 nm, and considered the values generated by the incubation of the factors and PBS (control) to be 100% activity.

### Intrinsic and extrinsic tenase complex assay

To evaluate whether CTX interferes in other activities of coagulation complexes, we assessed both extrinsic (TF/factor VII) and intrinsic (factor IX/factor VIII/PL/Ca^2+^) tenase complex activity using the Tissue Factor Human Chromogenic Activity Assay Kit (Abcam, Cambridge, UK) and the Biophen Factor IXa (Hyphen BioMed, Neuville-sur-Oise, France), respectively. 

Regarding the extrinsic tenase complex, CTX (0.5-13.5 µg/mL) or PBS (control) was incubated with human factor VII (FVII) and assay diluent for 10 minutes. Afterwards, recombinant human tissue factor lipoprotein (250 pM), FX and calcium were added and incubated for another 30 minutes. Subsequently, FXa chromogenic substrate was added and the reaction was monitored at 405 nm in Spectramax 190 microplate reader (Molecular Devices, San Jose, USA). All incubations and reaction procedures were performed at 37°C and followed the manufacturer’s instructions. 

To assay the intrinsic tenase complex, CTX (0.5-13.5 µg/mL) or PBS (control) was incubated with human factor IX (FIX) (1.5 µg/mL) (Sigma-Aldrich, St. Louis, USA) for 10 minutes. Afterwards, FX and factor VIII (FVIII) were added and incubated for 2 minutes, followed by addition of calcium and PL and incubated for 3 minutes. Subsequently, FXa chromogenic substrate was added and the reaction was monitored at 405 nm in Spectramax 190 microplate reader (Molecular Devices, San Jose, USA). All incubations and reaction procedures were performed at 37°C and followed the manufacturer’s instructions. 

The enzymatic activity was calculated based on the slope of the activity curve for each complex, which was obtained from sequential readings at 405 nm, considering 100% activity when factors were incubated with PBS (control).

### PBMC culture

Peripheral blood mononuclear cells were cultured in RPMI-1640 medium that was supplemented with penicillin/streptomycin (50 IU/mL and 50 μg/mL, respectively) and 10% fetal bovine serum (FBS), under an atmosphere of 5% CO_2_ at 37 °C. Cells were cultured in a 96-well plate (1x10^5^/well) for cell viability determination by MTT, quantification of cytokines and procoagulant activity (PCA). To evaluate cell viability by Annexin V/PI staining and TF, quantification cells were cultured in 24-well plate (5x10^5^ cells/well).

PBMCs were treated with lipopolysaccharide (LPS) (1 µg/mL), varying doses of CTX (0.04, 0.2 and 1 µg/mL) based on previous reports on the toxin’s anti-inflammatory and leukocyte function modulation activity [19-21], medium only (control) or CTX 30 minutes before addition of LPS. After the last treatment, the cultures were maintained for 24 hours, and afterwards the cell supernatant was collected and stored at -80°C for cytokine quantification. The remaining cells were assessed for procoagulant activity, cell viability (MTT and Annexin V/PI) and underwent TF quantification. 

### Whole blood culture

We also evaluated the anti-inflammatory/anticoagulant behavior of CTX in blood culture [22], using fresh whole blood collected in heparin. The experiment was performed on a 24-well plate using 500 µL of whole blood which was diluted (1:4 v/v) in DMEM complete medium (penicillin/streptomycin and 10% FBS). Blood was treated with LPS or LPS+CTX as described in section “PBMC culture”. After 24 hours of incubation under an atmosphere of 5% CO_2_ at 37 °C, supernatant was collected for cytokine quantification, the remaining blood was removed and PBMCs were isolated (as described in section “Whole blood, platelet poor plasma and PBMC”) for the procoagulant activity assay. 

### Cell viability

PBMC cell viability was performed using the MTT assay and Annexin V/PI staining, from cell culture treated with LPS (1 µg/mL) or CTX (0.04, 0.2 and 1 µg/mL). 

***MTT***

The MTT [3-(4,5-dimethylthiazol-2-yl)-2,5-diphenyltetrazolium bromide] assay was performed according to Mosmann [23], using a 96-well plate. The reaction absorbance was recorded at 570 nm using a Spectramax 190 microplate reader (Molecular Devices, San Jose, USA). Absorbance values for the control were considered as 100% of cell viability, and the results were expressed as a percentage (%) of viable cells.

***Annexin V/PI staining***

Cell viability was also evaluated using the apoptosis/necrosis kit (Invitrogen, Carlsbad, USA) containing Annexin V and propidium iodide (PI), according to the manufacturer’s instructions. The cells were cultured in 24-well plates for 24 hours as described above and then detached using Accutase (Sigma-Aldrich - St. Louis, USA), and transferred to FACS tubes on ice. Flow cytometry analyses were performed using a FACSCanto II flow cytometer equipped with the FACSDiva software (BD Biosciences, San Jose, USA), using 50,000 events for each sample.

### Cytokine and TF expression

The pro-inflammatory cytokines interleukin-1β (IL-1 β), interleukin-6 (IL-6), and tumor necrosis factor-α (TNF-α) were quantified from PBMC and whole blood culture supernatants using enzyme-linked immunosorbent assay (ELISA) kits, as recommended by the manufacturer (R&D Systems, Minneapolis, USA).

For TF quantification, PBMCs were detached using Accutase (Sigma-Aldrich, St. Louis, USA) and lysate by freezing and thawing 5 times. The TF quantification of cell lysate (20 µg protein/assay) was performed by ELISA using Human Coagulation Factor III/Tissue Factor DuoSet kit (R&D Systems, Minneapolis, USA), performed according to the manufacturer’s instructions. 

### Procoagulant activity assay

The PBMC procoagulant behavior was evaluated using the one-step plasma recalcification time assay [24]. After culture procedures, PBMCs and PBMCs from whole blood culture (1x10^5^/well in a 96-well plate) were washed with sterile PBS and incubated with 150 µL of human PPP at 37ºC for 10 min. Next, CaCl_2_ (30 mM) was added to the incubation mixture and plasma clotting was recorded using a Spectramax 190 microplate reader (Molecular Devices, San Jose, USA). The results were expressed as clotting time, determined as ½ of Vmax from the clotting curve of sequential readings at 405 nm.

### Binding assays

To assess the binding ability of CTX to PBMC, we used the toxin conjugated with fluorescein isothiocyanate (FITC) (Thermo Scientific, Rockford, USA), according to manufacturer's instructions. The unbound FITC was removed from FITC-CTX conjugate using a HiPrep 26/10 desalting column (GE Healthcare, Chicago, USA) on an AKTA FPLC system (GE Healthcare, Chicago, USA).

The binding assay was performed by incubating PBMC (1x10^6^ cells/tube) with PBS or different concentrations of FITC-CTX (12.5-100 µg/mL) for 30 minutes at 4°C in FACS tubes in a final volume of 300 µL of PBS. Then, the cells were washed with PBS and samples were analyzed by flow cytometry using FACSCanto II flow cytometer equipped with the FACSDiva software (BD Biosciences, San Jose, USA), from 50,000 events for each sample. The monocyte and lymphocyte population were distinguished based on FSC/SSC properties, and gating strategy protocol was performed as described in the literature [25], and illustrated in Additional file 1A. 

### Statistical analysis

Graphs were createdusing GraphPad Prism software version 5.01 (GraphPad Software In, San Diego, USA) and statistical analysis were then performed. A one-way analysis of variance (ANOVA) followed by a Dunnett post-test were used to analyze results after comparing groups. The unpaired Student’s t-test was used to analyze differences between the two data sets. Differences where p < 0.05 were considered statistically significant.

## Results

### CTX anticoagulant effects

***CTX mitigates coagulation in plasma and whole blood***

**Figure 1.**
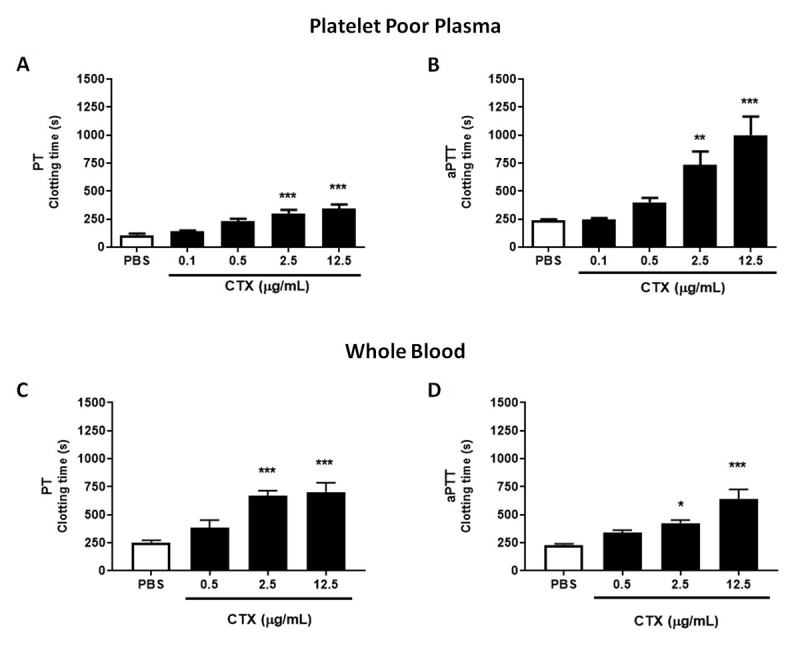
Whole blood and PPP clotting time. **(A** and **B)** PPP was incubated with CTX (0.1-12.5 µg/mL) or PBS (control). **(C** and **D)** Whole blood was incubated with CTX (0.5-12.5 µg/mL) or PBS (control), and PPP was obtained after incubation period. Clotting time was evaluated by **(A** and **C)** prothrombin time (PT) and **(B** and **D)** partial activated thromboplastin time (aPTT). The results are expressed as mean clotting time (s) ± SEM. The groups were performed with n = 6, where ***p < 0.001, **p < 0.01 and *p < 0.05 vs. control group. Statistical analysis was performed using one-way ANOVA followed by Dunnett’s post-test.

As observed in Figure 1, the toxin was effective in reducing PPP clot formation in both prothrombin time (PT - extrinsic pathway agonist) and partial activated thrombopastin time (aPTT - intrinsic pathway agonist) assays. Clot inhibition is observed from toxin concentration of 0.5 µg/mL, however statistically differently only from the concentration of 2.5 µg/mL, in a dose-response manner. Toxin also increased plasma clotting time from whole blood incubation, on both PT and aPTT assays, significantly from concentrations of 2.5 µg/mL. 

***CTX does not inhibit FXa and thrombin activity, but reduces prothrombinase complex activity at low concentrations of phospholipids***

**Figure 2.**
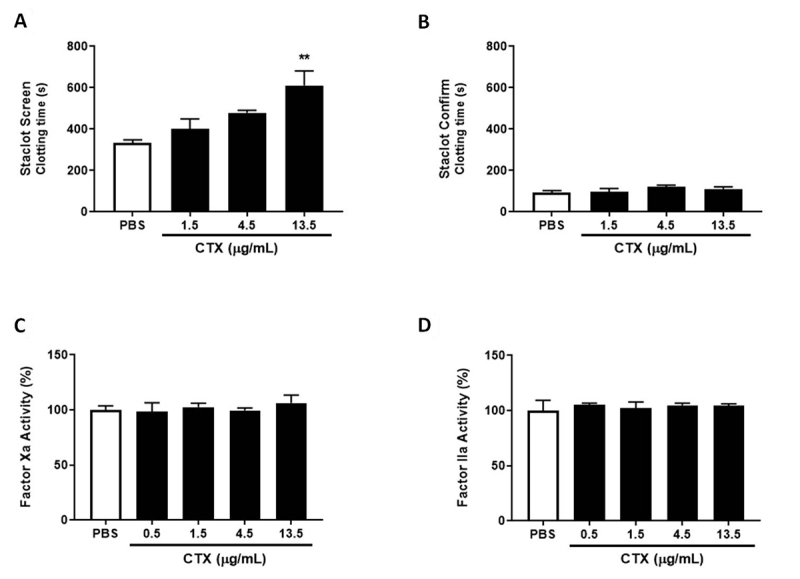
Staclot DRVV assay, FXa and thrombin activity. For the Staclot® DRVV assay, PPP was incubated with CTX (1.5-13.5 µg/mL) or PBS (control) for 10 min at 37°C, and afterwards **(A)** Staclot Screen or **(B)** Staclot Confirm reagents were added and coagulation time recorded. The results are expressed as mean clotting time (s) ± SEM. To evaluate the direct modulation of coagulation factors, CTX (0.5-13.5 µg/mL) or PBS (control) were incubated with **(C)** factor Xa or **(D)** thrombin and enzymatic activity. The results were expressed as coagulation factor activity (%) ± SEM, considering 100% activity when incubated with PBS (control). In both experiments the groups were performed with n = 4, **p < 0.01 vs. each respective control group. Statistical analysis was performed using one-way ANOVA followed by Dunnett’s post-test.

As observed in Figure 2A, CTX at a concentration of 13.5 µg/mL increased clotting time at low PL content. However, none of the CTX concentrations modulated clotting time at high PL content (Figure 2B). When the effects on isolated coagulation factors were evaluated, CTX did not modulate FXa or thrombin activity (Figure 2C and D), confirming in the present experiment that the toxin may interfere in the PLs’ role in prothrombinase complex activity and not in thrombin activity. 

***CTX impairs activity of both intrinsic and extrinsic tenase complexes***

**Figure 3.**
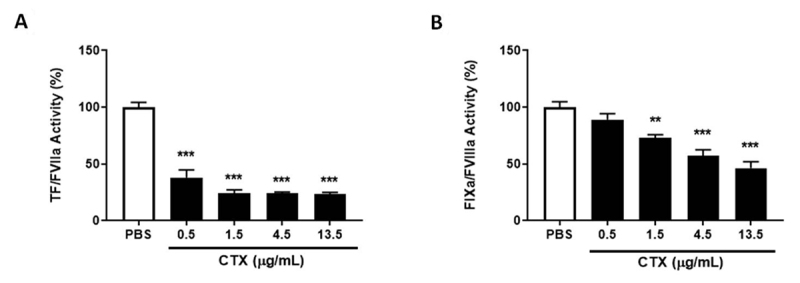
Intrinsic and extrinsic tenase complex activity. CTX (0.5-13.5 µg/mL) or PBS (control) were incubated with **(A)** TF/FVII or **(B)** FIX/FVIIIa/PL and FX activation measured. The results were expressed as complex activity (%) ± SEM, considering 100% activity when incubated with PBS (control). In both experiments the groups were performed with n = 5, ***p < 0.001 and **p < 0.01 vs. each respective control group. Statistical analysis was performed using one-way ANOVA followed by Dunnett’s post-test.

As observed in Figure 3A, CTX drastically decreased extrinsic complex activity in all evaluated concentrations, and reached an inhibition plateau of approximately 75% at a concentration of 1.5 µg/mL. As for the intrinsic complex, the toxin mitigated its activity in a dose-dependent manner, starting at the concentration of 1.5 µg/mL, and reaching ~54% inhibition at 13.5 µg/mL (Figure 3B). Considering that CTX does not interfere in FXa and thrombin activity alone (Figure 2C and D), the results obtained are strictly associated with the capacity of CTX to modulate the activity of intrinsic or extrinsic tenase complexes.

### Modulation of coagulation and inflammation cross talk by CTX

***CTX anti-inflammatory activity and inhibition of inflammation-induced coagulation: PBMC***

LPS, or the different concentrations of CTX used, did not alter PBMC viability, as evaluated by MTT and Annexin V/PI stain (Additional file 2). For evaluated inflammatory and coagulation parameters of PBMC culture, the experimental groups were consisted of cells treated with the medium (control), CTX, LPS or CTX + LPS. 

**Figure 4.**
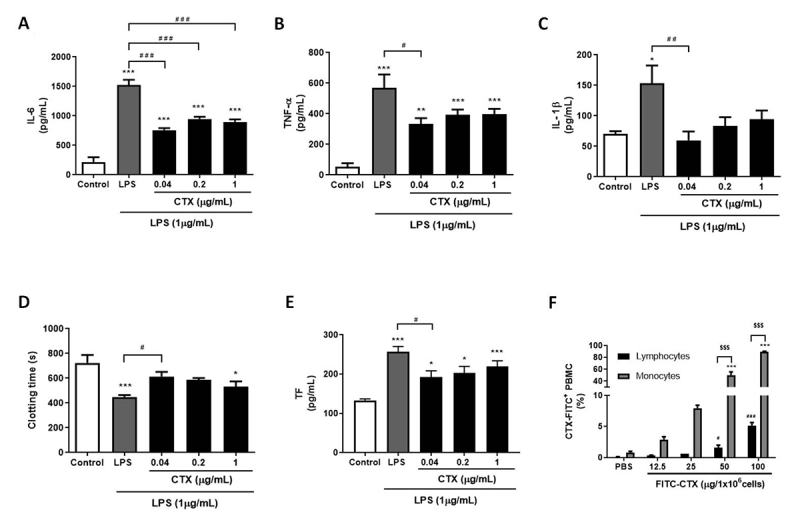
Anti-inflammatory, anticoagulant and cell binding behavior of CTX on PBMC. PBMC treated with medium only (control), LPS (1 µg/mL) or different concentrations of CTX (1, 0.2 or 0.04 µg/mL) 30 minutes before LPS addition, for 24 hours. From the cell supernatant, the pro-inflammatory cytokines **(A)** IL-6, **(B)** TNF-α and **(C)** IL-1β were quantified, and the remaining cells were submitted to the **(D)** procoagulant activity assay or cell lysate for **(E)** TF quantification. Results were expressed as mean cytokine concentration (pg/mL) ± SEM, mean clotting time (s) ± SEM and mean TF concentration (pg/mL) ± SEM. Experimental groups were performed with n = 6. ***p < 0.001, **p < 0.01 and *p < 0.05 vs. control, and ^###^p < 0.001, ^##^p < 0.01 and ^#^p < 0.05 vs. LPS. **(F)** The cell binding was evaluated by flow cytometry from PBMCs incubated with PBS only or FITC-CTX conjugate (12.5-100 µg/mL). The result was expressed as mean of FITC-CTX^+^ populations of monocytes and lymphocytes (%) ± SEM. Experimental groups were performed with n = 3. ***p < 0.001 vs. PBS (monocytes), ^###^p < 0.001 and ^#^p < 0.05 vs. PBS (lymphocytes) and ^$$$^p < 0.001 monocytes vs. lymphocytes (Student’s t-test). Statistical analysis was performed using one-way ANOVA followed by Dunnett’s post-test.

Regarding the inflammatory response, CTX alone (in any tested concentrations) did not stimulate the release of pro-inflammatory cytokines from PBMCs (Additional file 3A). However, when cells were previously incubated with the toxin before challenging with LPS, a reduction in IL-6 production was observed in all concentrations (Figure 4A). Although all concentrations of the toxin impaired TNF-α and IL-1β release from LPS-treated PBMCs, the concentration of 0.04 µg/mL was the only one which showed statistical significance (Figure 4B and C). 

In the procoagulant activity assay, PBMCs treated with LPS presented a procoagulant behavior and decreased the plasma clotting time when compared to the control group (Figure 4D). Previous treatment with CTX at 0.04 µg/mL mitigated the LPS-induced procoagulant behavior. This observation was consistent with the decrease in TF expression (Figure 4E). PBMCs in the presence of medium only (control), or treated with CTX, presented no difference in procoagulant behavior (Additional file 3B). 

The interaction to monocyte and lymphocyte from PBMCs was evaluated by flow cytometry using FITC-CTX. As illustrated in Figure 4F (and represented in Additional file 1B), the toxin was capable of binding to both leukocyte subsets at concentrations of 50 and 100 µg/mL, however with a remarkable binding preference for monocytes. 

***CTX anti-inflammatory activity and inhibition of inflammation-induced coagulation: whole blood***

**Figure 5.**
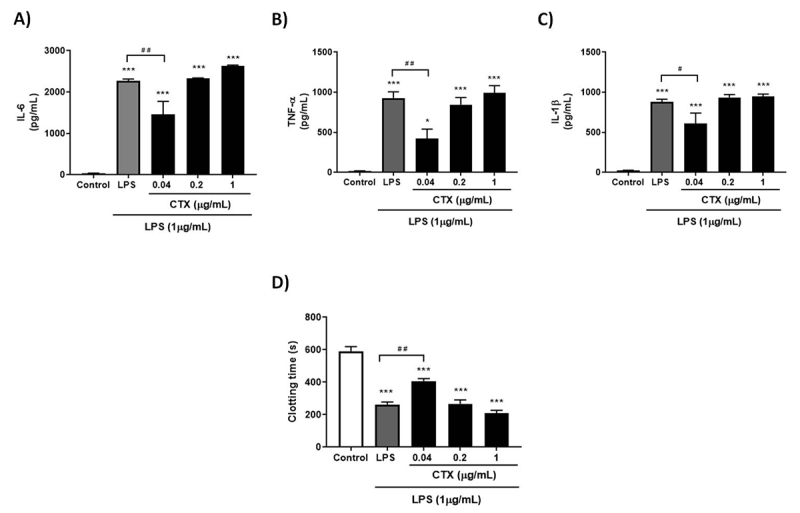
Anti-inflammatory and anticoagulant behavior of CTX on whole blood. Whole blood treated with medium only (control), LPS (1 µg/mL) or different concentrations of CTX (1, 0.2 or 0.04 µg/mL) 30 minutes before exposure to LPS, for 24 hours. From the supernatant, the pro-inflammatory cytokines **(A)** IL-6, **(B)** TNF-α and **(C)** IL-1β were quantified, and the remaining cells were submitted to the **(D)** procoagulant activity assay from the isolated PBMCs. Results were expressed as mean cytokine concentration (pg/mL) ± SEM and mean clotting time (s) ± SEM. Experimental groups were performed with n = 6. ***p < 0.001 and *p < 0.05 vs. control, and ^##^p < 0.01 and ^#^p < 0.05 vs. LPS. Statistical analysis was performed using one-way ANOVA followed by Dunnett’s post-test.

In WB culture, the same experimental groups were analyzed as for PBMCs. Differently from the isolated leukocytes, CTX showed a modest anti-inflammatory behavior, and a concentration of 0.04 µg/mL was found to be the only one capable of reducing IL-6, TNF-α and IL-1β production by LPS-stimulated WB (Figure 5A-C). 

The procoagulant activity was performed with the PBMCs obtained from the WB culture. PBMCs from blood treated with CTX at 0.04 µg/mL previous to treatment with LPS presented an increased plasma clotting time compared to LPS-treated blood, therefore a reduction in the PBMCs’ procoagulant behavior (Figure 5D).

## Discussion

CTX is considered a versatile toxin, since it is able to interact with a wide variety of biological targets and thus induces several physiological alterations [11]. In the present study, we have focused on investigating unknown anticoagulant mechanisms, as well as the modulation of inflammation and coagulation cross talk. 

The anticoagulant activity of CTX was initially described back in the 80’s and since then several other reports have revealed novel aspects regarding its mechanisms. It has been shown that both isolated CB and CA components, as well as CTX (which consists of CB+CA complex) are anticoagulant molecules, however with different potency (CB > CTX > CA) [12,26]. The results obtained in the present study showed that CTX significantly inhibited plasma clotting at 2.5 µg/mL in both in both PT and aPTT, with a clotting time fold increase (compare to control group) of 2.77 and 3.03, respectively,. Previous work by Souza [12] has also showed that CTX and its isolated components CB and CA inhibited plasma coagulation using PT and aPTT with an inhibitory efficiency stronger in the aPTT assay. We also evaluated the anticoagulant effects of CTX in whole blood, and found that the toxin increased clotting time in both PT and aPTT assays as observed in isolated plasma. This is the first report on CTX’s anticoagulant effect in whole blood, and shows that, even in a condition with several cellular and molecular targets [27], the toxin is still capable of having anticoagulant effects with high specificity.

The PT and aPTT assays are two of the most known and prescribed methods for evaluating coagulation abnormalities associated with alterations in the components of the intrinsic (PT), extrinsic (aPTT) and common (PT and aPTT) pathways [28]. Therefore, the results obtained in the present study and in previous reports [12,29] show that CTX inhibits both in the PT and the aPTT clotting assay, indicates that the toxin can act on different pathway components. Several efforts have been made to evaluate coagulation targets for svPLA_2._The prothrombinase complex (composed of FX, FVa and PL) have been shown to be an important target [10]. A study conducted by Faure [30] showed that the CTX interacts with FXa and mitigates the prothrombinase complex activity. The authors demonstrated that this inhibition was mediated by the CB subunit, and shows that the isoform CBc interact with FXa at high affinity and strongly inhibit the prothrombinase complex activity (without PL), while another isoform CBa2 also binds to FXa, with a weaker inhibition of the complex activity. On the other hand, the CA component does not interact with FXa or inhibit prothrombinase activity [30]. In the present study, we evaluated CTX effects on isolated FXa activity and found that the toxin did not alter the factor’s activity. Similar findings on other svPLA_2_s have been reported in the literature, showing their binding to FXa without interfering in FXa catalytic activity [31]. We also assessed the possible effects of CTX on coagulation PLs involving prothrombinase activity. We used the Staclot^®^ DRVV kit, which is used for the diagnosis of lupus anticoagulant, an antibody that binds to coagulant PLs and inhibits prothrombin complex formation and activity [32]. Our findings showed that CTX was only effective at inhibiting clot formation at low concentrations of PL and with lesser efficiency than in regular plasma, while in high concentrations of PL the toxin was ineffective. The results indicate that CTX anticoagulation activity is sensitive to low PL concentrations, resulting in a prolonged clotting time, while the high concentrations neutralize the toxin and abolish the inhibition. Mounier et al. [9] have shown that CB inhibited 80% of prothrombinase activity in the absence of phospholipid, and only 20% in its presence. These results show that the inhibitory effects of CTX on prothrombin activity involve the modulation of coagulant PLs, possibly being associated with the hydrolysis of these phospholipids or by the competition for phospholipid binding to coagulation factors, as observed in other svPLA_2_s [10].

Aside from the prothrombinase complex, we also evaluated the modulation of both intrinsic and extrinsic tenase complexes by CTX, and observed that the toxin inhibited both pathways. Previous reports of this nature have also been published for other svPLA_2_s such as daboxin P, from *Daboia russelii* snake venom, and 3-finger toxins such as exactin, from *Hemachatus haemachatus* venom, in which both toxins inhibit extrinsic and intrinsic tenase complexes [33,34]. Moreover, our data shows that CTX is more efficient in inhibiting the intrinsic tenase complex than the extrinsic, similarly to that observed for daboxin P and exactin. Considering that the activity of both the tenases and prothrombinase complexes critically depends on the phospholipid composition, the binding preference of CTX to different phospholipids could be associated with its efficiency to inhibit both tenase complexes distinctively [35,36].

The inflammation-induced coagulopathy represents an important issue in diseases with an inflammatory background, such as sepsis. In this situation, the participation of TF is a hallmark event which is responsible for procoagulant effects that lead to hemorrhagic and thrombotic disorders [14]. Tissue factor is a transmembrane glycoprotein that forms the extrinsic tenase complex with FVIIa, and is expressed mostly by endothelial cells and monocytes through different stimuli such as pathogenic agents and their isolated components, such as LPS, as well as inflammatory mediators [37]. CTX has been described as exhibiting an anti-inflammatory response by impairing inflammatory stimulation of leukocytes [6]. We evaluated the modulation of the inflammation-induced coagulation by assessing inflammatory cytokines and procoagulant behavior of cells from PBMC and whole blood culture stimulated with LPS. The endotoxin is the main component of Gram-negative bacteria’s cell wall and has pro-inflammatory properties. LPS stimulate the production of several inflammatory cytokines from leukocytes via toll-like receptor activation, and is widely applied in septic shock experimental models [38]. 

Our results show that CTX impairs the production of the pro-inflammatory cytokines IL-6, TNF-α and IL-1β from both PBMC and whole blood cultures. Several studies have demonstrated the anti-inflammatory behavior of CTX by using different inflammatory agonists and models. In the case of LPS, Freitas [21] reported that CTX was capable of mitigating the production of IL-12, TNF-α and IL-6, as well as cell surface activation markers from dendritic cells treated with LPS. Moreover, De Andrade [39] demonstrated that the toxin reduced the inflammatory mediators IL-6, IL-1β, IL-8, nitric oxide and PGI_2_ and the adhesion molecules E-selectin, ICAM and VCAM from human umbilical vein endothelial cells (HUVEC) challenged with LPS. We also observed that CTX presents anti-inflammatory and anticoagulant properties in whole blood, indicating that the toxin interacts with a wide variety of cellular and soluble targets in blood (such as leukocytes and coagulation components) and modulated their function.

The anti-inflammatory effect of CTX was followed by a reduction of PBMC procoagulant behavior that was induced by TF. The toxin reduced the procoagulant activity of PBMCs (from PBMC and whole blood cultures) and impaired the increased expression of TF by LPS. Since our results show that CTX bind preferentially to monocytes compared to lymphocyte, and that monocytes are more avid in expressing TF [40], it’s possible to assume that CTX inhibitory effects are associated with a direct action on monocytes. Although snake venoms and their isolated components have been described as increasing the expression of TF associated with a pro-inflammatory response [41-43], this is the first report of a venom toxin which inhibits its expression via an anti-inflammatory activity. A previous study by Andrade [44] demonstrated that CTX modulated the production of molecules involved in thrombogenesis by LPS-treated HUVEC, inducing a reduction of von Willebrand factor (platelet aggregating factor) and plasminogen activator inhibitor-1 (antifibrinolytic agent) and an increase in protein C (anticoagulant properties) and tissue plasminogen activator (fibrinolytic agent). The elucidated mechanism involving the immunomodulatory effects of CTX is associated with the toxin’s capacity to induce the expression of lipoxin A_4_ (LXA_4_), a pro-resolving lipid mediator with anti-inflammatory and immunomodulatory properties thru formyl peptide receptors (FPRs) activation [6]. Although in the present study we did not focus on the toxin’s mechanism, it has been found that 15-epi-lipoxin A_4_ reduces TF expression in TNF-α treated HUVECs [45], and is therefore possibly responsible for CTX’s ability to reduce inflammation-induced coagulation. 

Another interesting fact observed in the present study is that CTX at the lowest concentration evaluated (0.04 µg/mL) was the most efficient concentration inducing anti-inflammatory behavior in PBMC and whole blood, and reduced PBMC procoagulant activity, and TF expression. Other reports on CTX immunomodulatory effects (such as leukocyte spreading and phagocytic activity), for which experimental procedures were performed using different concentrations of the toxin, have shown a slight tendency to decrease its beneficial activity in higher concentrations of CTX [19,20,46]. A possible explanation could be associated with the desensitization of receptors involved in CTX’s effects. [47]. When a receptor is desensitized, by prolonged stimulation or high concentrations of the agent (in this case CTX or products derived from CTX activity), the cell becomes refractory to further stimulation [48]. The desensitization of FPRs by LXA_4_ and analogs have been described [49], and could be associated with the phenomena of lack of anti-inflammatory effects of high concentrations of CTX.

## Conclusion

The present study investigated the anticoagulant effects of CTX and its capacity to impair inflammation-induced coagulation. The toxin presented a direct effect on coagulation by mitigating the prothrombinase complex activity by modulating the role of the coagulation phospholipids. In addition, the toxin decreased both intrinsic and extrinsic tenase complexes activity. As for the inflammation-coagulation cross talk, the toxin showed an anti-inflammatory effect, followed by a reduction in PBMC procoagulant activity due a decrease in tissue factor expression. Due its anticoagulant and anti-inflammatory properties, the results establish the toxin as a possible pharmacological strategy in coagulopathies with inflammatory background, such as sepsis and other similar conditions.

### Abbreviations

aPTT: partial activated thromboplastin time; Cdt: *Crotalus durissus terrificus*; CTX: crotoxin; DIC: disseminated intravascular coagulation; FBS: fetal bovine serum; FITC: fluorescein isothiocyanate; FITC-CTX: crotoxin conjugated to fluorescein isothiocyanate; FIX: factor IX; FIXa: factor IX activated; FVII: factor VII; FVIIa: factor VII activated; FVIII: factor VIII; FVIIIa: factor VIII activated; FX: factor X; FXa: factor X activated; HUVEC: human umbilical vein endothelial cells; IL-12: interleukin 12; IL-1β: interleukin 1β; IL-6: interleukin 6; IL-8: interleukin 8; LPS: lipopolysaccharide; LXA4: lipoxin A4; MTT: 3-(4,5-dimethylthiazol-2-yl)-2,5-diphenyltetrazolium bromide; PBMC: peripheral blood mononuclear cells; PBS: phosphate buffer saline; PI: propidium iodide; PL: phospholipids; PPP: platelet poor plasma; 

PT: prothrombin time; svPLA_2_: snake venom phospholipases A_2_; TF: tissue factor; TNF-α: tumor necrosis factor α; WB: whole blood.

## Supplementary material

The following online material is available for this article:

Additional file 1. FITC-CTX binding to PBMCs. PBMCs were incubated with FITC-CTX conjugate (12.5-100 µg/mL) and submitted to flow cytometry analysis. **(A)** Gate strategy to differentiate monocyte and lymphocyte populations in PBMCs. **(B)** Histogram representation of monocytes and lymphocytes labeled with FITC-CTX.

Additional file 2. Cell viability. PBMC were treated with medium only (control) LPS (1 µg/mL) or CTX (1, 0.2 and 0.04 µg/mL) for 24 hours and cell viability was assessed. **(A)** In the MTT assay, the results are expressed by the mean of cell viability (%) ± SEM. The Annexin V/PI staining cell viability was represented by **(B)** viable cells (% Annexin V- PI- population) ± SEM and **(C)** dot plot flow cytometry representation, each quadrant defining cell state (viable cells AV−PI−; cells in apoptosis AV+PI−; cells in necrosis AV−PI+; or cells in late apoptosis AV+PI+) and their percentage. MTT assay experimental groups were performed with n = 6, whereas AnnexinV/PI with n = 3. Statistical analysis was performed using one-way ANOVA followed by Dunnett’s post-test.

Additional file 3. Effects of CTX for PBMC procoagulant activity and cytokine production only. PBMCs treated with medium only (control) or different concentrations of CTX (1, 0.2 and 0.04 µg/mL) for 24 hours. Afterwards, cell supernatant was collected to quantify **(A)** the pro-inflammatory cytokines IL-6, TNF-α and IL-1β; and cells were submitted to the **(B)** procoagulant activity assay. Results were expressed as mean cytokine concentration (pg/mL) ± SEM and clotting time (s) ± SEM. Statistical analysis was performed using one-way ANOVA followed by Dunnett’s post-test.
