# Supplementary material for: Role of crotoxin in coagulation: novel insights into anticoagulant mechanisms and impairment of inflammation-induced coagulation
Source: J Venom Anim Toxins Incl Trop Dis. 2020 Nov 27;26:e20200076. doi: 10.1590/1678-9199-JVATITD-2020-0076 (PMC7702976; doi:10.1590/1678-9199-JVATITD-2020-0076)
Supplement: Additional file 1. [file 1678-9199-jvatitd-26-e20200076-s1.pdf]

J Venom Anim Toxins incl Trop Dis
https://doi.org/10.1590/1678-9199-JVATITD-2020-0076

Supplementary Material to "Role of crotoxin in coagulation: novel insights into anticoagulant mechanisms and impairment of inflammation-induced coagulation."

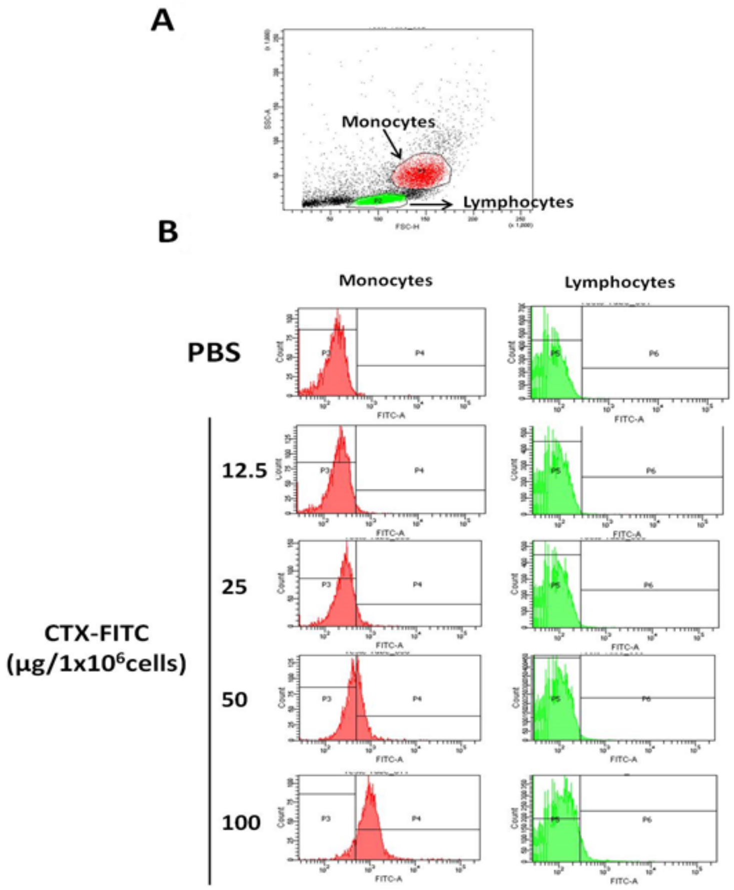

Additional file 1. FITC-CTX binding to PBMCs. PBMCs were incubated with FITC-CTX conjugate (12.5-100 µg/mL) and submitted to flow cytometry analysis. **(A)** Gate strategy to differentiate monocyte and lymphocyte populations in PBMCs. **(B)** Histogram representation of monocytes and lymphocytes labeled with FITC-CTX.
