# Supplementary material for: Role of crotoxin in coagulation: novel insights into anticoagulant mechanisms and impairment of inflammation-induced coagulation
Source: J Venom Anim Toxins Incl Trop Dis. 2020 Nov 27;26:e20200076. doi: 10.1590/1678-9199-JVATITD-2020-0076 (PMC7702976; doi:10.1590/1678-9199-JVATITD-2020-0076)
Supplement: Additional file 2. [file 1678-9199-jvatitd-26-e20200076-s2.pdf]

J Venom Anim Toxins incl Trop Dis
https://doi.org/10.1590/1678-9199-JVATITD-2020-0076

Supplementary Material to "Role of crotoxin in coagulation: novel insights into anticoagulant mechanisms and impairment of inflammation-induced coagulation."

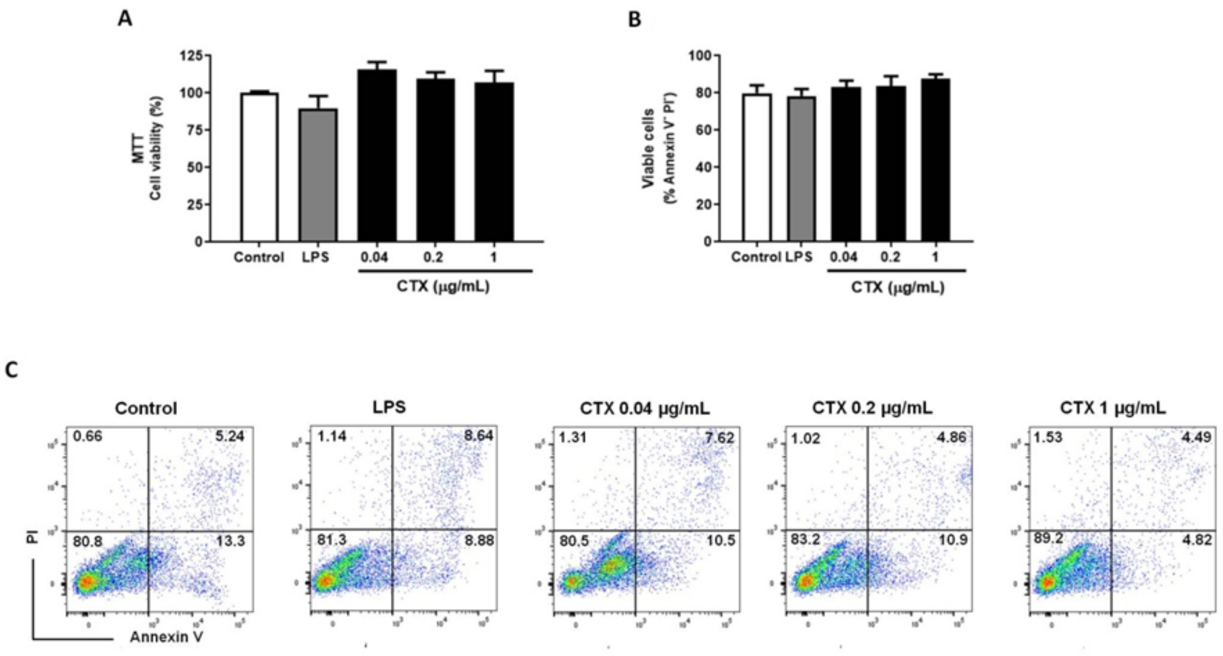

Additional file 2. Cell viability. PBMC were treated with medium only (control) LPS (1 µg/mL) or CTX (1, 0.2 and 0.04 µg/mL) for 24 hours and cell viability was assessed. **(A)** In the MTT assay, the results are expressed by the mean of cell viability (%) ± SEM. The Annexin V/PI staining cell viability was represented by **(B)** viable cells (% Annexin V- PI- population) ± SEM and **(C)** dot plot flow cytometry representation, each quadrant defining cell state (viable cells AV−PI−; cells in apoptosis AV+PI−; cells in necrosis AV−PI+; or cells in late apoptosis AV+PI+) and their percentage. MTT assay experimental groups were performed with n = 6, whereas AnnexinV/PI with n = 3. Statistical analysis was performed using one-way ANOVA followed by Dunnett's post-test.
