# Supplementary material for: Role of crotoxin in coagulation: novel insights into anticoagulant mechanisms and impairment of inflammation-induced coagulation
Source: J Venom Anim Toxins Incl Trop Dis. 2020 Nov 27;26:e20200076. doi: 10.1590/1678-9199-JVATITD-2020-0076 (PMC7702976; doi:10.1590/1678-9199-JVATITD-2020-0076)
Supplement: Additional file 3. [file 1678-9199-jvatitd-26-e20200076-s3.pdf]

J Venom Anim Toxins incl Trop Dis
https://doi.org/10.1590/1678-9199-JVATITD-2020-0076

Supplementary Material to "Role of crotoxin in coagulation: novel insights into anticoagulant mechanisms and impairment of inflammation-induced coagulation."

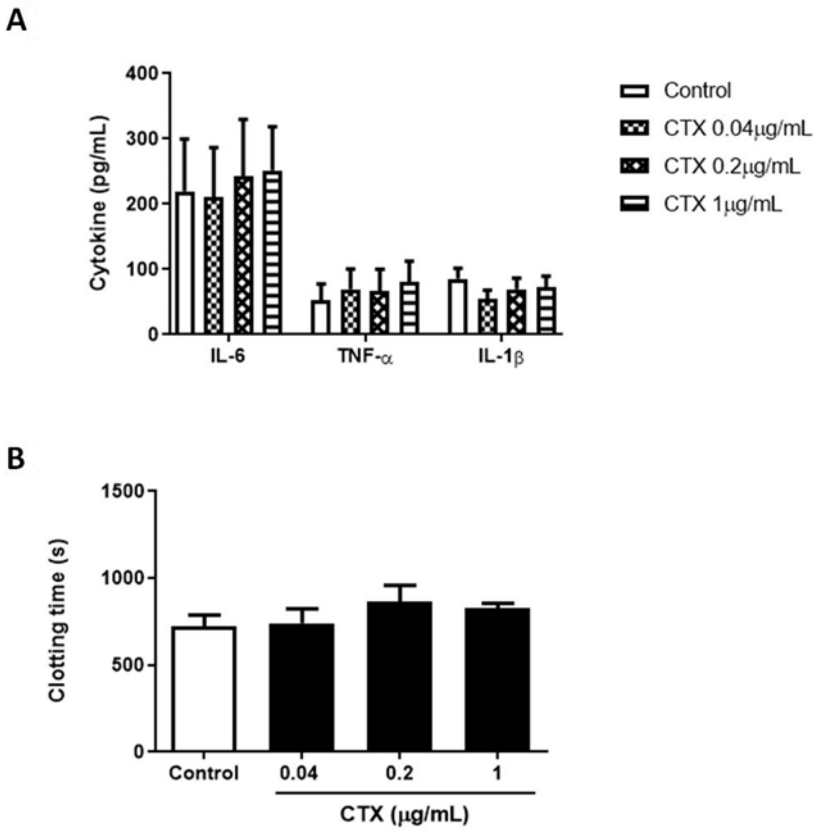

Additional file 3. Effects of CTX for PBMC procoagulant activity and cytokine production only. PBMCs treated with medium only (control) or different concentrations of CTX (1, 0.2 and 0.04 μg/mL) for 24 hours. Afterwards, cell supernatant was collected to quantify **(A)** the pro-inflammatory cytokines IL-6, TNF-α and IL-1β; and cells were submitted to the **(B)** procoagulant activity assay. Results were expressed as mean cytokine concentration (pg/mL) ± SEM and clotting time (s) ± SEM. Statistical analysis was performed using one-way ANOVA followed by Dunnett's post-test.
